# Genome-wide characterization and expression analysis of LBD transcription factors in *Ziziphus jujuba* var. *spinosa*: putative roles in tissue development and abiotic stress adaptation

**DOI:** 10.3389/fpls.2025.1602440

**Published:** 2025-05-21

**Authors:** Meng Li, Qiaoyun Zhang, Shuhui Zhou, Ruixue Wang, Yao Zhao, Jibiao Geng

**Affiliations:** ^1^ Shandong Provincial Key Laboratory of Water and Soil Conservation and Environmental Protection, College of Resources and Environment, Linyi University, Linyi, Shandong, China; ^2^ Shandong Provincial Forestry Protection and Development Service Center, Department of Natural Resources of Shandong Province, Jinan, Shandong, China

**Keywords:** genome-wide analysis, expression patterns, stress response, LBD, sour jujube

## Abstract

The plant-specific Lateral Organ Boundaries Domain (LBD) transcription factors (TFs) are critical regulators of the expression of genes related to tissue development and stress responses. Sour jujube (*Ziziphus jujuba* var. *spinosa*), a stress-tolerant rootstock for jujube cultivation, remains understudied in terms of its *LBD* gene family. In this study, a genome-wide analysis of the sour jujube genome was performed and *37 ZjLBD* genes were identified. These genes were phylogenetically classified into two classes and six subgroups based on evolutionary relationships with *Arabidopsis* and poplar. These genes were unevenly distributed across 11 chromosomes, with 15 segmental duplication events detected. Upstream TFs of *ZjLBD*s were predicted and grouped into six interactive networks, revealing that the functions of *ZjLBD*s may be enriched in stress response, hormone signaling, and tissue development. RNA-seq data demonstrated tissue-specific expression patterns of *ZjLBD*s, with limited genes highly expressed in fruit. The *ZjLBD23* may regulate the development of flowers and white mature fruits, while *ZjLBD11, ZjLBD13, ZjLBD22* and *ZjLBD28* may be involved in lateral root formation. Analysis of the cis-acting elements, homology relationships and abiotic stress-induced expression levels suggested functional divergence: *ZjLBD13*, *ZjLBD19* and *ZjLBD28* may contribute to extreme cold resistance, *ZjLBD11*, *ZjLBD14*, *ZjLBD33* and *ZjLBD35* may be associated with drought tolerance, and *ZjLBD9*, *ZjLBD13* and *ZjLBD22* with salt stress adaptation. This study provides critical insights into the biological roles of *ZjLBD*s and lays a foundation for breeding sour jujube varieties with enhanced stress resilience.

## Introduction

Global climate change has precipitated a surge in extreme weather events, subjecting plants to unprecedented challenges from drought, salinity, and freezing stresses. To survive under these adverse conditions, plants deploy intricate physiological and molecular adaptations. Central to these adaptive strategies are the molecular mechanisms governing stress perception, signal transduction, and transcriptional reprogramming—processes critically orchestrated by transcription factors (TFs), for example WRKY, NAC and bZIP, that regulate stress-responsive gene networks (Zhang et al., 2022). These proteins typically possess DNA-binding domains that enable sequence-specific interactions with cis-acting elements in promoter regions, thereby modulating transcriptional activity. Among plant-specific TF families, the Lateral Organ Boundaries Domain (LBD) proteins, also termed ASymmetric Leaves 2-like (ASL), stand out as unique regulators exclusive to higher plants. Characterized by their conserved LOB domain, LBD TFs coordinate diverse developmental processes and stress adaptation mechanisms, ranging from organ morphogenesis to abiotic stress responses (Husbands et al., 2007; Majer and Hochholdinger, 2011).

Since the initial identification of 42 *LBD* genes in *Arabidopsis thaliana* (Shuai et al., 2002), numerous plant genome have been sequenced, revealing the presence of LBD TF family members in various species. These include 47 LBDs in tomato (Gupta and Gupta, 2021); 58 in apple (Wang et al., 2013); 40 in grape (Cao et al., 2016) and 57 in poplar (Zhu et al., 2007). Characterized by three structural domains extending from the N- to C-terminus, LBD TFs feature a zinc finger-like C-block (CX2CX6CX3C) for DNA binding, a Gly-Ala-Ser block (GAS block) associated with key LBD functions, and a leucine-like zipper module (LX6LX3LX6L). These domains collectively enable LBD TFs to regulate diverse biological processes, including plant organ development (roots, shoot meristems, leaves, flowers, and embryos), tissue regeneration, nitrogen metabolism, hormonal signaling, and stress responses (Fan et al., 2012).

LBD TFs exhibit functional conservation in regulating root development across diverse plant species (Omary et al., 2022). For instance, the TFs WOX11 and LBD16, in conjunction with the histone demethylase JMJ706, collectively orchestrate the development of crown roots in rice (Geng et al., 2024). Similarly, AtLBD16 and AtLBD29 have been identified as key regulators of lateral root formation (Okushima et al., 2007). Beyond root development, LBDs play pivotal roles in various plant tissues. *AtLBD6* controls the stem meristem (Uchida et al., 2007), specifies leaf adaxial identity (Xu et al., 2003), and influences sepal and petal development (Xu et al., 2008). *AtLBD30* is implicated in embryogenesis and flower development (Borghi et al., 2007; Soyano et al., 2008). In poplar, *PtaLBD1* regulates secondary phloem development (Yordanov et al., 2010), while the ectopic expression of *EgLBD37* and *EgLBD29* promotes the differentiation of secondary xylem and the production of phloem fibers (Lu et al., 2018). In addition, LBD TFs also feature prominently in nitrogen metabolism, hormone signaling, and stress tolerance. Studies have shown that *Arabidopsis thaliana LBD37/38/39* and *Malus domestica MdLBD13* negatively regulate anthocyanin biosynthesis, and nitrogen uptake and assimilation (Rubin et al., 2009; Li et al., 2017). Furthermore, the downstream *LBD* genes of auxin response factors, namely *LBD16*, *LBD17*, *LBD18*, and *LBD29*, are rapidly activated by callus-inducing medium and the ectopic expression of these genes can autonomously induce callus formation without exogenous hormones (Fan et al., 2012). *AtLBD1/3/4/11* in *Arabidopsis thaliana* mediates the balance of cytokinin-induced secondary growth (Ye et al., 2021). In contrast, the Class II *LBD* gene family member *ZmLBD5* in maize negatively regulates the production of abscisic acid (ABA) and gibberellins, thereby impacting drought tolerance (Feng et al., 2022). Furthermore, the overexpression of *VvLBD39* reduces the tolerance of grape to drought and salt stress by regulating stomatal closure, promoting transpiration, and decreasing the capacity for reactive oxygen species scavenging (Chen et al., 2024); whereas the ZAT6-LBD16 transcriptional module regulates lateral root development under salt stress by promoting downstream cell wall remodeling, which could benefit plant adaptation to salt stress (Zhang et al., 2024).

The sour jujube (*Ziziphus jujuba* var. *spinosa*), believed to be the ancestor of common jujube, is valued for its health-promoting properties. Its leaves are used for crafting tea, and jujuboside extracted from its seeds is a crucial component in functional beverages, and insomnia treatments. Meanwhile, the sour jujube is distinguished by its extensive and deep root system, which confers remarkable drought tolerance and the ability to thrive in nutrient-poor soils. These characteristics make it an ideal candidate for cultivation in barren mountains. With over 900 varieties of jujube, the resistance of the tree to various stresses largely depends on the rootstock used. Sour jujube is commonly used as the rootstock for jujube trees due to its remarkable tolerance to drought and poor soils (Liu et al., 2020b). Previous studies have identified an autotetraploid sour jujube and shown that its unique xylem morphology contributes to its exceptional drought tolerance. Notably, several *LBD* genes were identified in the downstream screening for xylem development-related ZjVND7 (Li et al., 2021, 2022). However, the roles of these *LBD* genes under abiotic stress remain unclear. In this study, *LBD* genes in sour jujube will be identified, and their gene structure, protein motif structure, cis-acting elements in promoters, chromosomal localization, and the phylogenetic relationships will be analyzed. Additionally, RNA-seq and quantitative real-time PCR (qRT-PCR) analysis will elucidate the expression patterns of these genes during tissue development and under abiotic stresses such as high salinity, drought, and low temperatures. This research is anticipated to elucidate the functional roles of the *LBD* gene family in sour jujube and establish a foundation for comprehending the mechanisms underlying organogenesis and stress responses.

## Materials and methods

### Identification of *LBD* genes in sour jujube

The *LBD* gene family members in sour jujube were identified using two methods. Initially, 43 *LBD* genes from *Arabidopsis thaliana* and 57 *LBD* genes from poplar were downloaded from the *Arabidopsis* Information Resource (TAIR, http://www.Arabidopsis.org/) and the Phytozome database (http://rice.plantbiology.msu.edu/), respectively. A blastp search (E < 1 × 10^-5^) was conducted against the Telomere-to-Telomere (T2T) genome of sour jujube (Li et al., 2024) using the 43 *Arabidopsis LBD* genes as queries. Subsequently, the Hidden Markov Model (HMM) file for the LOB domain (PF03195) was downloaded from the Pfam database (http://pfam.sanger.ac.uk/), and the sour jujube LBD protein sequences were obtained using the HMMER program (version 3.2.1). The program was run with default settings and a cutoff value of 10^-5^. Finally, an intersection of the LBD proteins identified by both methods was taken, retaining only those protein sequences encoded by the longest transcripts of the same gene. The LOB domain in ZjLBDs were verified using InterPro (http://www.ebi.ac.uk/interpro/) and SMART (http://smart.embl-heidelberg.de/) according to Letunic et al. (2012).

### The domain, phylogeny, and classification analysis of *LBD* in sour jujube

Six AtLBD and six PtLBD sequences were selected as reference sequences from different subgroups. The full-length LBD protein sequences in sour jujube were aligned using DNAMAN (version 6.0) to delineate the LOB domain. A phylogenetic tree was constructed using the neighbor-joining method with 1000 replicates in MEGA (version X). Based on phylogenetic relationships, the LBD proteins in sour jujube were categorized into distinct classes and subgroups, following the classification methods of AtLBD (Shuai et al., 2002) and PtLBD (Zhu et al., 2007).

### Properties and structural analysis of *LBD* proteins in sour jujube

The open reading frame (ORF) length, molecular weight (MW), and isoelectric point (pI) of LBD proteins in sour jujube were calculated using the ExPASy website (http://web.expasy.org/protparam/). The conserved motifs of LBD proteins in sour jujube were identified using MEME (version 5.1.0, http://meme-suite.org/tools/meme), with the maximum number of motifs set to 20 (Bailey et al., 2009).

### Chromosome localization and gene structure analysis of *ZjLBD*s

The chromosomal localization of *ZjLBD* genes was determined using MapInspect (http://www.softsea.com/review/MapInspect.html) and aligned to the genomic chromosomes. The selective pressure on *ZjLBD* genes was assessed through Ka/Ks ratios. The exon-intron structure of *ZjLBD* genes was predicted by comparing the coding sequence (CDS) with their corresponding full-length DNA sequences using the Gene Structure Display Server (http://gsds.cbi.pku.edu.cn) (Hu et al., 2015). Visualization was performed using TBtools (version 1.0) (Chen et al., 2020).

### Analysis of cis-regulatory elements in the *ZjLBD*s promoters

A 2.0-kb sequence upstream of the transcription start site of *ZjLBD* was extracted from the sour jujube genome and submitted to the PlantCARE website (http://bioinformatics.psb.ugent.be/webtools/plantcare/html/) to identify cis-regulatory elements involved in hormone signaling, defense and stress responses, and growth and development regulation (Lescot et al., 2002).

### Regulatory network and GO enrichment analysis of *ZjLBD*s and its upstream transcription factors

To identify the potential TF genes binding with *ZjLBD*s promoters, the promoter sequences of *ZjLBD*s were retrieved. Following the method described by Tian et al. (2020), the binding site prediction tool available on the PlantRegMap platform (https://plantregmap.gao-lab.org/index.php) we utilized to scan for TF binding sites with a threshold p-value ≤ 1e^-4^. The potential TF genes identified through this process were further subjected to Gene Ontology (GO) enrichment analysis. GO terms with a p-value < 0.01 were selected as significantly enriched terms.

### Expression patterns of *ZjLBD*s in diverse tissues

To investigate the expression patterns of *ZjLBD* genes across various tissues, RNA-seq datasets from 9 distinct tissue types—namely root, leaves, flower, stem, branch, young fruit, white mature fruit, half-red fruit, and fully red fruit—were retrieved from the National Center for Biotechnology Information (NCBI) database under Sequence Read Archive (SRP046073) (Liu et al., 2014). After filtering the raw data, SRA files were converted to FASTQ format through SRA Toolkit (version 2.8.2). Quantitative gene expression analysis was conducted using Cufflinks (version 2.2.1) on the aligned read files, with gene expression levels being determined based on the Fragments Per Kilobase per Million mapped reads (FPKM).

### Plant materials and stress treatments

To further confirm the differential expression levels of *ZjLBD* genes identified in RNA-seq in different tissues, samples of root, leaves, flower, stem, branch, and fruit were collected from three ‘Dongzao’ jujube trees for experiments in 2024.

To analyze the expression levels of *ZjLBD* genes under abiotic stresses, 45-day-old rooted sour jujube seedlings were transferred to Hoagland nutrient solution for 1 week and then subjected to simulated drought and salt conditions. For drought treatment, seedlings were placed in a 20% PEG6000 solution, and leaves were collected after 6 h, 12 h, and 48 h of treatment. For salt treatment, seedlings were placed in Hoagland nutrient solution containing 150 mmol/L NaCl, and leaves were collected after 24 h and 48 h of treatment. Untreated seedlings served as control group for both drought and salt treatments.

For cold stress, branches with consistent growth vigor were selected from three Dongzao jujube trees. These branches were then exposed to temperatures of 4 °C, -10°C, -20°C, -30°C, and -40°C for 10 h at a cooling rate of 5 °C/h, and the branches treated at 4 °C were used as control samples. Then the xylem of branches was collected.

All experiments were conducted with three biological replicates. After collection, samples were flash-frozen in liquid nitrogen and stored at -80°C for qRT-PCR analysis.

### RNA extraction and qRT-PCR analysis

RNA extraction was performed using the Aidlab RNA Extraction Kit (Aidlab Biotechnologies Co., Ltd, Beijing, China). Following quantification and integrity assessment of the extracted RNA, qRT-PCR analysis was conducted using a Roche Light Cycler 96 instrument with the 2^-ΔΔCt^ method (Livak and Schmittgen, 2001). The primers used are listed in **Supplementary Table 1**.

## Results

### Identification, location and property analysis of *ZjLBD* genes

A combination of two bioinformatics approaches was employed to identify members of the *LBD* gene family in sour jujube. Initially, we performed a BLASTP search using 43 LBD TFs from *Arabidopsis thaliana* as queries, identifying 38 candidate LBD proteins in sour jujube. Subsequently, we utilized a HMM based on the LOB domain (Pfam PF03195) to identify 38 LBD proteins. By intersecting the results from both methods and removing redundant sequences, we ultimately identified 37 unique *LBD* genes for further analysis (**Table 1**).

**Table 1:** Identification, localization and property analysis of *ZjLBD* genes and ZjLBD proteins in sour jujube.

Name	Gene ID	RefSeq ID	Location	Strand	ORF (aa)	CDS (bp)	MW (Da)	pI
*ZjLBD1*	LOC107410445	XM_016017882.2	chr1:3798302.3799186	Plus	294	885	32520.4	9.17
*ZjLBD2*	LOC107414156	XM_016022255.3	chr1:6669095.6671478	Minus	269	810	30286.2	6.66
*ZjLBD3*	LOC107414165	XM_048467305.1	chr1:6673443.6676416	Minus	293	882	32440.2	6.91
*ZjLBD4*	LOC107418709	XM_016027414.3	chr1:13089585.13091788	Minus	172	519	18710.4	8.8
*ZjLBD5*	LOC107425243	XM_016035197.3	chr1:46143303.46144513	Plus	259	780	29475.2	5.73
*ZjLBD6*	LOC125421142	XM_048469049.1	chr2:36705.40013	Plus	228	687	24832.7	8.55
*ZjLBD7*	LOC107411328	XM_016018894.3	chr2:12492933.12494798	Plus	215	648	23205.4	5.76
*ZjLBD8*	LOC107411547	XM_016019153.3	chr2:17951612.17952381	Plus	173	522	19233.8	6.94
*ZjLBD9*	LOC107405098	XM_016012108.3	chr2:18459614.18461975	Minus	234	705	25323.7	9.01
*ZjLBD10*	LOC112490873	XM_025071312.2	chr3:9989030.9990361	Minus	234	705	25911.9	5.08
*ZjLBD11*	LOC107413601	XM_016021596.3	chr3:13261675.13263740	Plus	232	699	25283.9	6.7
*ZjLBD12*	LOC107413901	XM_016021956.3	chr3:20781450.20782143	Minus	201	606	22060	7.55
*ZjLBD13*	LOC107413913	XM_016021966.3	chr3:20963808.20964867	Plus	184	555	20704.4	6.02
*ZjLBD14*	LOC107413969	XM_016022041.3	chr3:21388777.21390380	Minus	231	696	25123.5	8.99
*ZjLBD15*	LOC107415475	XM_016023814.3	chr4:2068051.2070247	Plus	175	528	19210.3	6.99
*ZjLBD16*	LOC107435643	XM_025068029.2	chr4:3207081.3208476	Minus	333	1002	37545	6.14
*ZjLBD17*	LOC107416542	XM_016025045.3	chr4:6796731.6798364	Plus	306	921	33215.6	8.22
*ZjLBD18*	LOC107417180	XM_048472789.1	chr4:13582150.13584259	Plus	232	699	25898	8.76
*ZjLBD19*	LOC107418072	XM_016026742.3	chr5:6556918.6560997	Minus	169	510	18520.1	8.18
*ZjLBD20*	LOC107418298	XM_048474199.1	chr5:9485227.9487936	Plus	197	594	21232.8	6.89
*ZjLBD21*	LOC125422406	XM_048473930.1	chr5:10494452.10495852	Plus	303	912	34121.9	5.67
*ZjLBD22*	LOC107418786	XM_016027481.3	chr5:15087860.15089191	Plus	261	786	27855.4	8.52
*ZjLBD23*	LOC107420556	XM_016029544.3	chr6:2990959.2992413	Minus	212	639	23020.2	8.05
*ZjLBD24*	LOC107420167	XM_016029063.3	chr6:3888361.3889941	Minus	250	753	28303.2	7.15
*ZjLBD25*	LOC107422222	XM_016031644.2	chr7:11887018.11888447	Minus	155	468	17720.9	8.71
*ZjLBD26*	LOC107423945	XM_016033602.2	chr8:2909733.2911211	Minus	245	738	28035.6	6.31
*ZjLBD27*	LOC107423646	XM_016033233.3	chr8:4828094.4829101	Plus	196	591	21919.9	5.79
*ZjLBD28*	LOC107435688	XM_016047315.3	chr8:5644662.5647820	Minus	245	738	25139.4	7.65
*ZjLBD29*	LOC107405081	XM_016012088.3	chr8:5658346.5661120	Plus	205	618	22549.9	8.83
*ZjLBD30*	LOC107406151	XM_016013254.3	chr8:10599663.10603433	Minus	316	951	34976	5.97
*ZjLBD31*	LOC107425917	XM_016035977.3	chr9:4348087.4350651	Plus	205	618	22452.5	8.34
*ZjLBD32*	LOC107425834	XM_025076976.2	chr9:4358211.4359074	Minus	231	696	25612.7	5.88
*ZjLBD33*	LOC107425833	XM_016035881.3	chr9:4369009.4369908	Minus	237	714	26048.9	6.11
*ZjLBD34*	LOC125424336	XM_048481496.1	chr9:13105437.13106474	Minus	206	621	22590.6	5.52
*ZjLBD35*	LOC107429450	XM_016040135.3	chr10:22844726.22846093	Plus	252	759	26803.1	8.46
*ZjLBD36*	LOC107405321	XM_016012356.3	chr12:14231037.14232518	Plus	253	762	27466.7	6.35
*ZjLBD37*	LOC107424931	XM_016034826.3	chrNW_025964593.1:111674.113249	Minus	238	717	25753.2	8.57

**Figure 1:**
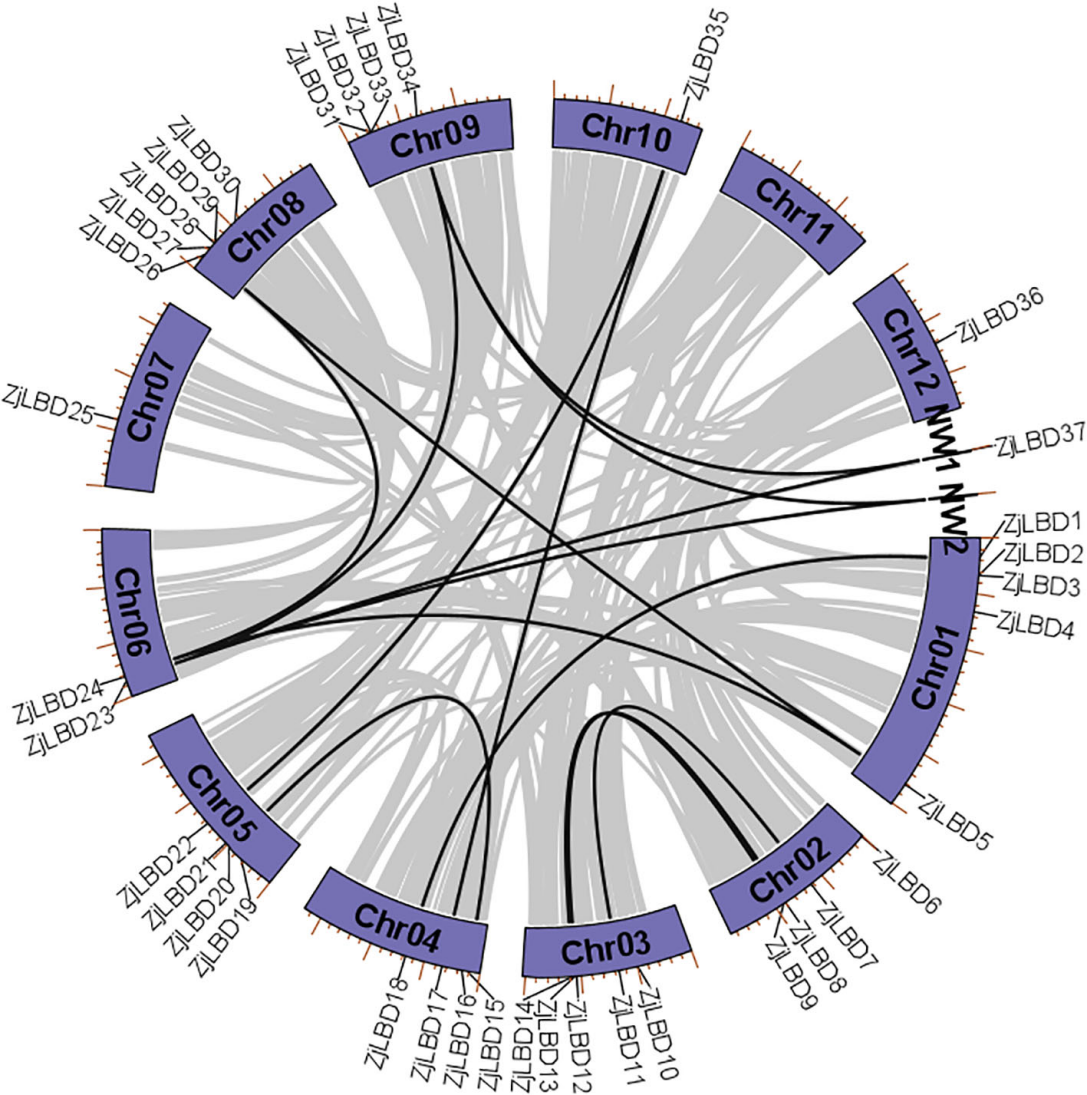
Chromosome distribution and collinearity analysis of *ZjLBD* genes.

Based on the positional information of *LBD* genes on chromosomes, the 37 *LBD* genes were designated as *ZjLBD1*-*ZjLBD37*. A comprehensive analysis was conducted on these genes and their encoded proteins, encompassing gene location, ORF length, CDS length, MW, and pI. As shown in **Table 1** and **Figure 1**, *ZjLBD*s are distributed across 11 chromosomes. The CDS lengths range from 468 bp (ZjLBD25) to 1002 bp (ZjLBD16), while the ORF lengths of the LBD proteins vary from 155 amino acids (ZjLBD25) to 333 amino acids (ZjLBD16). The MW of the proteins span from 17.72 kDa (ZjLBD25) to 37.55 kDa (ZjLBD16), and the predicted pI range from 5.08 (ZjLBD10) to 9.17 (ZjLBD1).

Fifteen homologous gene pairs were detected among nine chromosomes in the *ZjLBD*s, representing inter-chromosomal collinearity events. Notably, *ZjLBD23* located on Chr6 and *ZjLBD34* on Chr9 were identified as high-frequency genes for collinearity events, each occurring three times.

To assess the selective pressure on these colinear *ZjLBD* genes during the evolutionary process, the ratio of the number of nonsynonymous substitutions per nonsynonymous site (Ka) to the number of synonymous substitutions per synonymous site was analyzed (**Supplementary Table 2**). The results indicated that for 13 colinear events involving 19 *ZjLBD* genes, 11 pairs of *ZjLBD* genes exhibited Ka/Ks ratios less than 1, suggesting that these genes were under purifying selection.

### Phylogenetic analysis and domain recognition of *ZjLBD* family proteins

**Figure 2:**
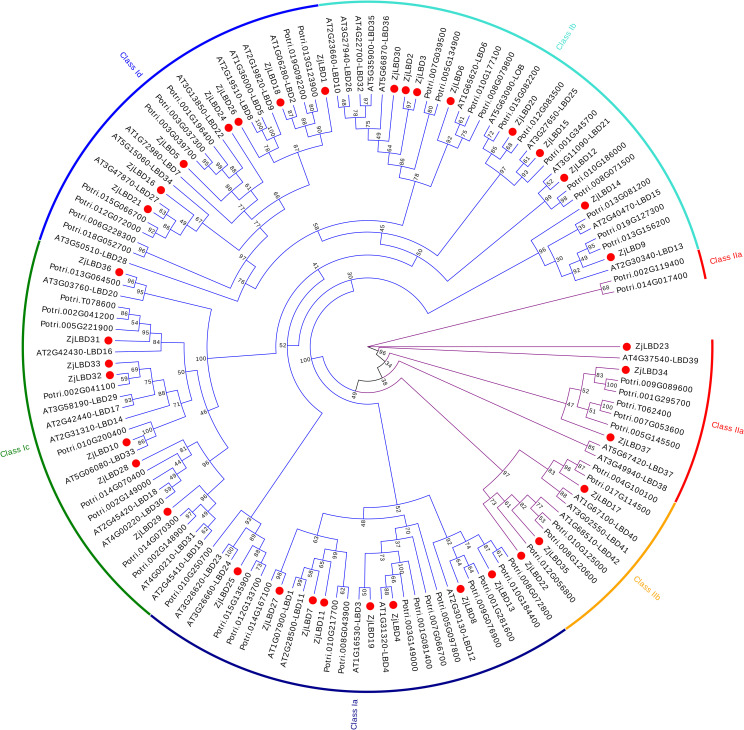
Phylogenetic analysis of LBD proteins in *Arabidopsis thaliana*, poplar and sour jujube.

A phylogenetic analysis was conducted on 43 *Arabidopsis* and 57 poplar LBD proteins along with the LBD family proteins of sour jujube (**Figure 2**). These proteins were classified into two major groups, Class I and Class II, which were further divided into four (subgroups Ia to Id) and two subgroups (subgroups IIa to IIb), respectively. Class I contained 31 ZjLBD proteins, while Class II included only 6. Similarly, the number of LBD proteins in *Arabidopsis* and poplar was higher in Class I than in Class II.

**Figure 3:**
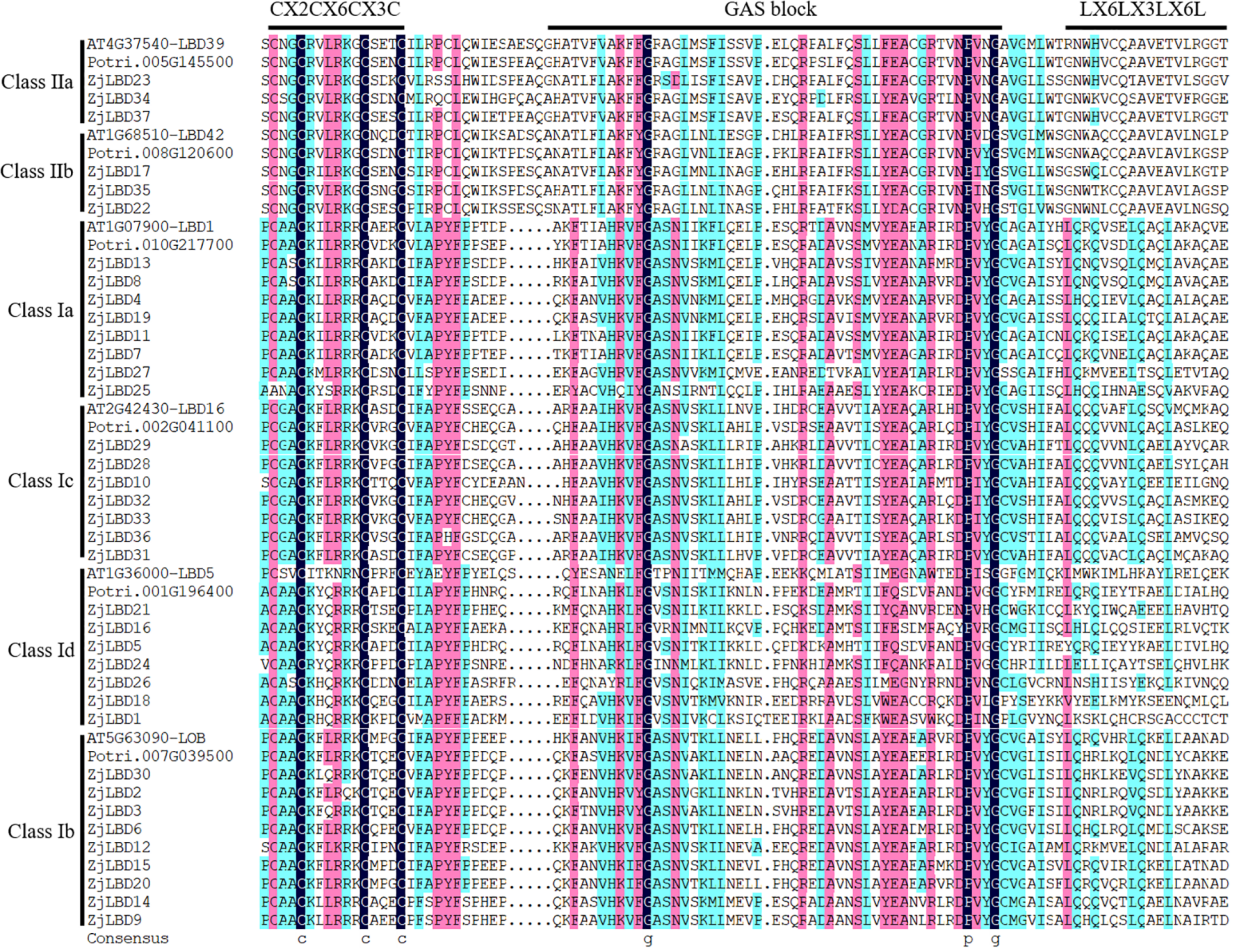
The structural domains of ZjLBD proteins.

Six AtLBD proteins (AT4G37540-LBD39, AT1G68510-LBD42, AT1G07900-LBD1, AT2G42430-LBD16, AT1G36000-LBD5, AT5G63090-LOB) and six PtLBD proteins (Potri.005G145500, Potri.008G120600, Potri.010G217700, Potri.002G041100, Potri.001G196400, Potri.007G039500) were randomly selected from the six subgroups. Their LOB domains were used as references to identify the domains in ZjLBDs. As shown in **Figure 3**, all 37 ZjLBD proteins possess a conserved N-terminal motif composed of cysteine residues (CX2CX6CX3C) indicating their DNA-binding capabilities. The C-terminus of Class I contains a leucine zipper-like motif (LX6LX3LX6L), which is associated with protein dimerization. Additionally, subgroups Ia, Ib, and Ic contain a conserved glycine-alanine-serine (GAS) domain near the C-terminus, which includes conserved proline and glycine residues.

### Analysis of *ZjLBD*s encoding protein motif and gene structure

**Figure 4:**
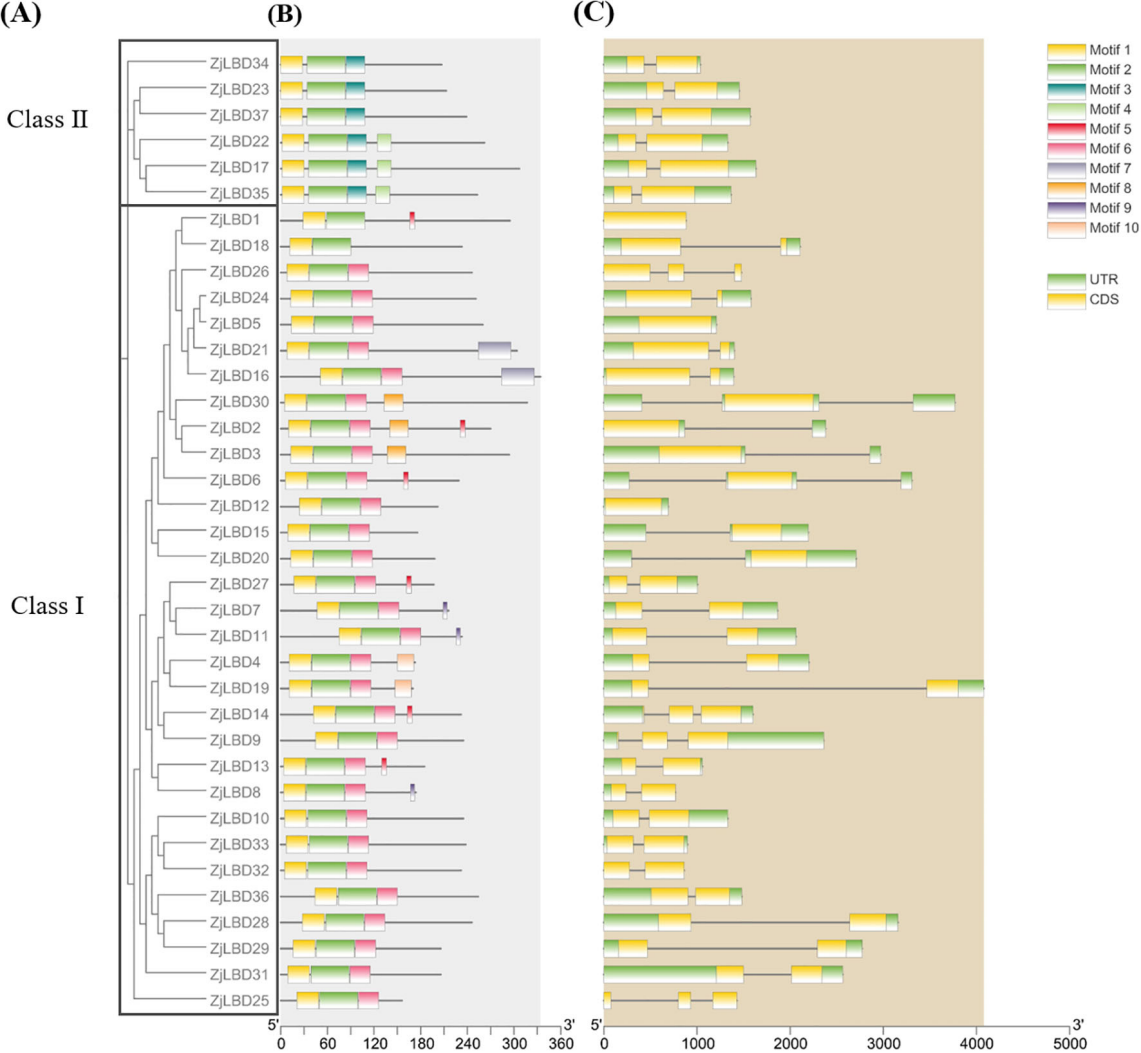
Phylogeny **(A)**, putative motif analysis **(B)**, and gene structure **(C)** of each ZjLBD protein and *ZjLBD* gene. Exon and intron are represented by yellow boxes and grey lines, respectively. Lengths of exons and introns of *ZjLBD*s are displayed proportionally.

Structural analysis of the *ZjLBD* genes and their encoded proteins revealed distinct motif compositions and gene structures. As depicted in **Figures 4A, B**, ZjLBD proteins contain 10 motifs, with motif 1 and motif 2 consistently present at the N-terminus. Class I and Class II proteins exhibit different motif compositions, with motif 3 being unique to Class II. Notably, all Class I proteins, except for ZjLBD1 and ZjLBD18, contain motif 6. Additionally, unique motifs (motifs 5, 7, 8, 9, and 10) are found in only 2 to 6 ZjLBD proteins within Class I, suggesting that these proteins may have distinct functions.

The coding and non-coding regions of the *ZjLBD* genes were also analyzed. The lengths of all *ZjLBD* genes were within 4500bp, with the numbers of coding region varying from one (*ZjLBD1*, *ZjLBD2*, *ZjLBD3*, *ZjLBD5*, *ZjLBD6*, *ZjLBD12*, *ZjLBD15*, *ZjLBD20*, *ZjLBD30*) to three (*ZjLBD25*, *ZjLBD26*). As shown in **Figure 4C**, the structures of Class I and Class II genes were distinctly different, with more than half of the *ZjLBD* genes in Class I exhibiting longer intron sequences compared to those in Class II.

### Analysis of cis-acting elements in the promoters of *ZjLBD* genes

**Figure 5:**
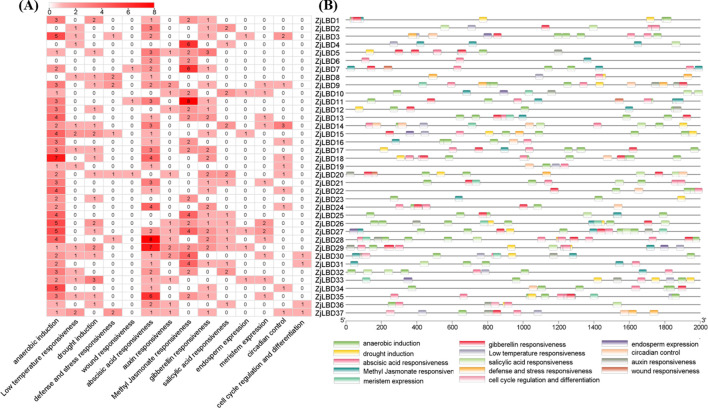
The number **(A)** and distribution **(B)** of cis-acting elements in the promoters of *ZjLBD* genes.

A total of 100 types of cis-acting elements were detected in the *ZjLBD*s promoters, including 8 unnamed elements, which were annotated as having 43 functions. The TATA-box was the most frequently detected element, present in all *ZjLBD*s promoters. For further analysis, 14 types of functional annotations were selected. As shown in **Figure 5**, the anaerobic induction element (ARE) and the abscisic acid response element (ABRE) were present in all *ZjLBD*s. Specifically, 8, 7, and 6 ABREs were found in the promoters of *ZjLBD28*, *ZjLBD29*, and *ZjLBD35*, respectively. At least one of the five hormone response elements was present in each *ZjLBD* promoter, and eight methyl jasmonate responsiveness elements have been identified in the promoter of *ZjLBD11*. Notably, drought induction element coexisted with ABRE in 14 *ZjLBD*s promoters. Of the 13 *ZjLBD*s promoters containing auxin response elements, four types were identified: AuxRR-core, TGA-element, TGA-box, and AuxRE. Except for *ZjLBD5* and *ZjLBD12*, all of these *ZjLBD*s promoters co-occur with elements annotated in growth-related pathways. The promoters of 24 *ZjLBDs* contain 6 types of development-related elements associated with cell cycle regulation, seed-specific expression, meristem and endosperm development, and circadian rhythm control, with meristem expression-related element identified in 11 of these promoters. Additionally, 9 types of site-binding elements were identified in the *ZjLBD*s promoters, among which the MYB binding site involved in drought-inducibility, was the most common. These findings suggest that *ZjLBD* genes promoters harbor cis-acting elements associated with hormone responsiveness, stress induction, and growth-related functions, highlighting their potential roles in stress adaptation and developmental regulation.

### Upstream TFs of *ZjLBD*s associated with stress responses and tissue development

**Figure 6:**
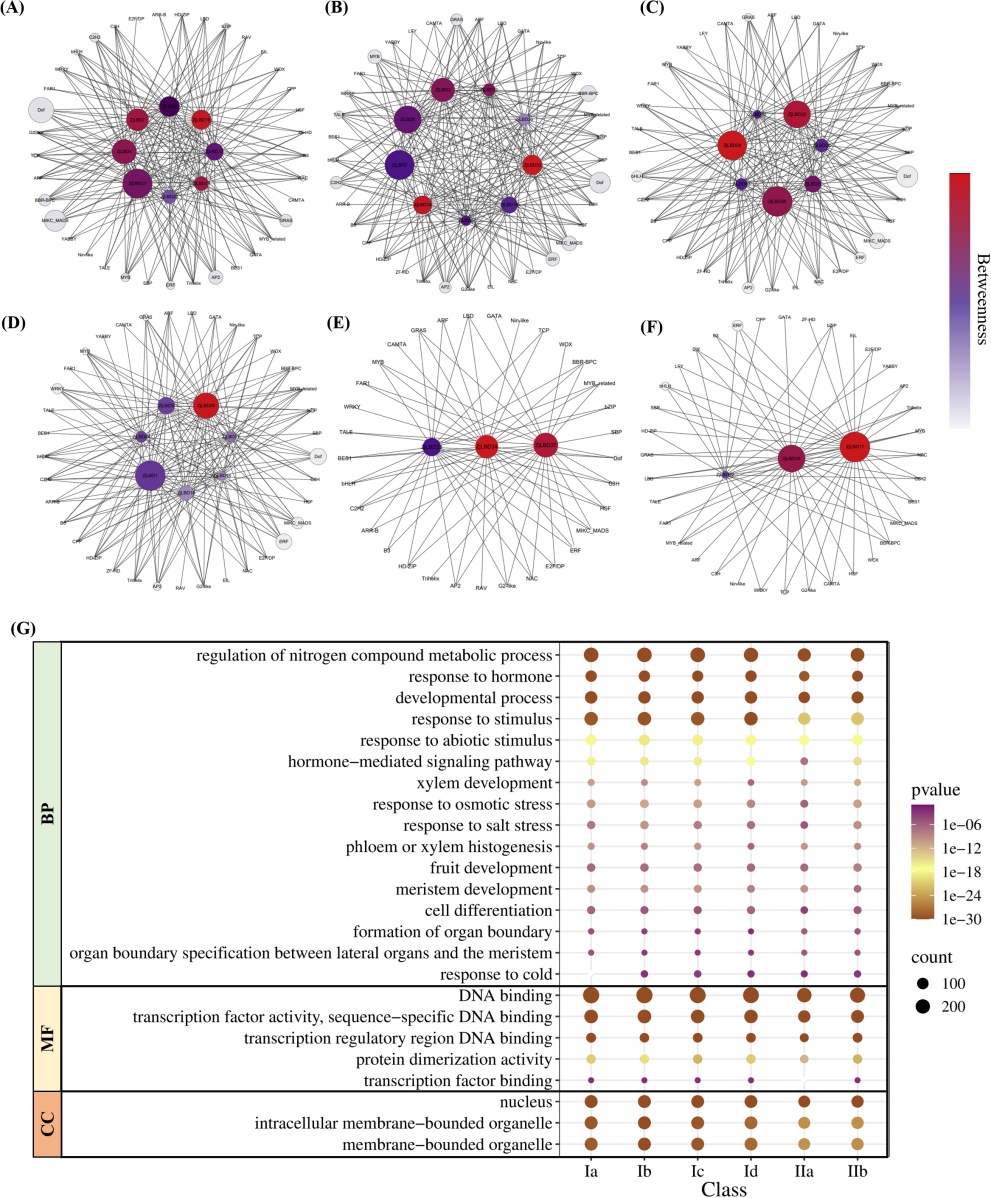
Regulatory network and GO enrichment analysis of *ZjLBD*s and its upstream transcription factors. **(A-F)** Regulatory network of *ZjLBD*s and its potential upstream TFs in subgroups Ia **(A)**, Ib **(B)**, Ic **(C)**, Id **(D)**, IIa **(E)**, IIb **(F)**. **(G)** GO enrichment analysis of potential upstream TFs genes of *ZjLBD*s in six subgroups.

We conducted a comprehensive analysis of transcriptional regulatory sites in the promoters of *ZjLBD*s, and identified 350 potential upstream genes encoding 39 types of TFs that collectively have 11,261 binding sites across the promoter sequences of 37 *ZjLBD*s. Among these *ZjLBD*s, the promoter of *ZjLBD36* was predicted to possess the highest number of TF binding sites, totaling 516, and it may interact with 166 upstream TF genes. The Dof has the most binding sites with the *ZjLBD*s promoters. Additionally, the highest numbers of genes encoding ERF and MYB were predicted, with 44 and 41 genes, respectively. As shown in **Figures 6A–F**, the interaction regulatory network analysis of upstream TF binding to the promoters of *ZjLBD*s in six subgroups revealed that subgroup Ia is centered around *ZjLBD19*, which is likely regulated by 87 genes encoding TFs. Subgroup Ib includes *ZjLBD14* and *ZjLBD30*, which may be regulated by 103 and 112 TF genes, respectively. Subgroup Ic is represented by *ZjLBD29*, regulated by 187 TF genes, while subgroup Id is represented by *ZjLBD26*, regulated by 172 TF genes. Subgroup IIa is centered around *ZjLBD34*, regulated by 147 TF genes, and subgroup IIb is centered around *ZjLBD17*, regulated by 189 TF genes. These findings reveal the intricate and diverse transcriptional regulatory modules of ZjLBDs across different subgroups, providing a basis for elucidating their functional divergence.

The TF genes potentially binding to the *ZjLBD*s promoters were subjected to Gene Ontology (GO) enrichment analysis, revealing that 342 TF genes were enriched in 415 GO terms (**Figure 6G**). The GO enrichment analysis indicated that the upstream TF genes of *ZjLBD*s in each subgroup were significantly enriched in the Biological Process (BP) terms regulation of nitrogen compound metabolic process and response to stimulus. Notably, the enriched *p*-values for response to stimulus and response to abiotic stimulus in the upstream TF genes of *ZjLBD* genes in Class II were higher than those in Class I. Meanwhile, the annotation count of upstream TF genes in response to osmotic stress and response to salt stress was lower in Class II than in Class I, yet the number remains greater than 18. Except for subgroup Ia, which lacked upstream gene annotations for response to cold, all other subgroups had more than 10 genes annotated in this pathway. Additionally, approximately 10 genes were annotated in pathways related to xylem development and phloem or xylem histogenesis. Five subgroups on average possess 27, 19 and 4 upstream TF genes annotated in fruit development, meristem development, and formation of organ boundary. In the Molecular Function (MF) category, the most abundant term was DNA binding. Significant differences were observed between Class I and II in terms of protein dimerization activity and specific DNA-binding functions. Specifically, subgroup IIa has more than 40 fewer genes annotated in transcription factor activity, sequence-specific DNA binding compared to Class I, and no genes were annotated in transcription factor binding. Finally, no significant differences were observed in the Cellular Component (CC) annotations of the potential upstream genes across the six subgroups. Therefore, although the upstream TF genes of *ZjLBD*s in different subgroups exhibit similar functions, these factors form modules that may exhibit hierarchical or intensity-dependent characteristics in tissue development and stress adaptation.

### Expression patterns of *ZjLBD*s in various tissues

**Figure 7:**
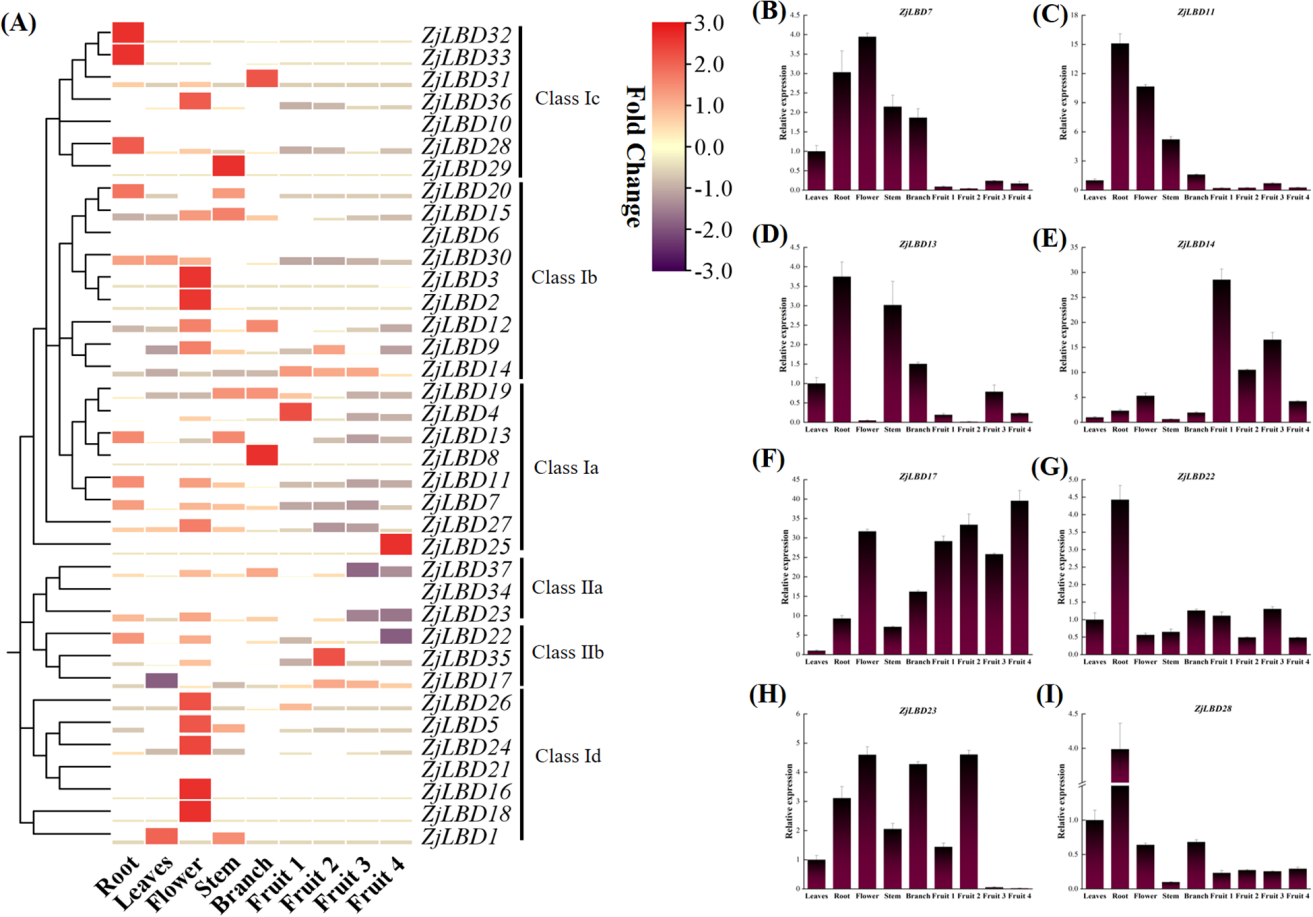
Expression pattern of *ZjLBD* genes in root, leaves, flower, stem, branch and fruit of sour jujube. **(A)** Expression patterns of *ZjLBD*s from RNA-seq data. The height of the bars represents the fold change of gene expression. **(B-I)** qRT-PCR analysis of selected differentially expressed *ZjLBD* genes, including *ZjLBD7* **(B)**, *ZjLBD11* **(C)**, *ZjLBD13* **(D)**, *ZjLBD14* **(E)**, *ZjLBD17* **(F)**, *ZjLBD22* **(G)**, *ZjLBD23* **(H)** and *ZjLBD28* **(I)**. Fruit 1: Young fruit, Fruit 2: White mature fruit, Fruit 3: Semi-red fruit, Fruit 4: Fully red fruit.

Based on publicly available RNA-seq data, the expression levels of *ZjLBD* genes were analyzed across six different tissues: root, leaves, flower, stem, branch and fruit (**Figure 7A**). The results revealed that different *ZjLBD*s showed distinct expression patterns across various plant tissues. Notably, 11 *ZjLBD* genes from subgroups Ia, Ib, Id, and IIa exhibited higher expression levels in flowers compared to other tissues. The root tissue contained 7 highly expressed *ZjLBD* genes, including *ZjLBD*7 (subgroup Ia) and *ZjLBD*22 (subgroup IIb), whose expression levels were 312-fold (for semi-red fruits) and 16-fold (for fully red fruits) higher than those in the least expressing tissue, respectively. Furthermore, *ZjLBD12* and *ZjLBD23* were highly expressed in flowers, whereas *ZjLBD15* was predominantly expressed in stems, and *ZjLBD19* and *ZjLBD37* were highly expressed in branches. Notably, there was almost no high expression of *ZjLBD* genes in leaves. The expression levels of *ZjLBD* genes in jujube fruits were generally low, with only 6 *ZjLBD* genes exhibiting FPKM values exceeding 20. When comparing the expression of *ZjLBD* genes across four developmental stages of fruit, 8 *ZjLBD* genes from five subgroups showed elevated expression during the white mature stage. These *ZjLBD* genes included members from subgroup IIb, as well as *ZjLBD11* (subgroup Ia) and *ZjLBD37* (subgroup IIa).

To validate the expression levels of these genes across different tissues, *ZjLBD7*, *ZjLBD11*, *ZjLBD13, ZjLBD14*, *ZjLBD17*, *ZjLBD22*, *ZjLBD23* and *ZjLBD28* were further selected for qRT-PCR analysis (**Figures 7B–I**). The expression level of *ZjLBD17* was significantly lower in leaves, while it was relatively higher in fruits. In contrast, *ZjLBD7* and *ZjLBD11* exhibited lower expression levels in fruits compared to in other tissues. Notably, *ZjLBD11*, *ZjLBD13*, *ZjLBD22*, and *ZjLBD28* had higher expression levels in roots, while *ZjLBD7* and *ZjLBD23* were more highly expressed in flowers. During the four developmental stages of fruit development, *ZjLBD14* showed a higher expression level in young fruit, where it reached the highest expression, with the maximum fold change being 45 times higher than that in stems. Meanwhile, *ZjLBD13*, *ZjLBD14*, and *ZjLBD23* exhibited distinct differential expression patterns. Specifically, *ZjLBD13* and *ZjLBD14* showed a pattern of initial decrease, followed by an increase, and then a decrease again, while *ZjLBD23* demonstrated a pattern of initial increase followed by a sharp decline, with its expression level dropping 84-fold from the white mature stage to the semi-red stage. The expression patterns of these genes in different tissues reveal their potential roles in the development of specific tissues.

### Expression patterns of *ZjLBD*s under low temperature, drought, and salt stress

**Figure 8:**
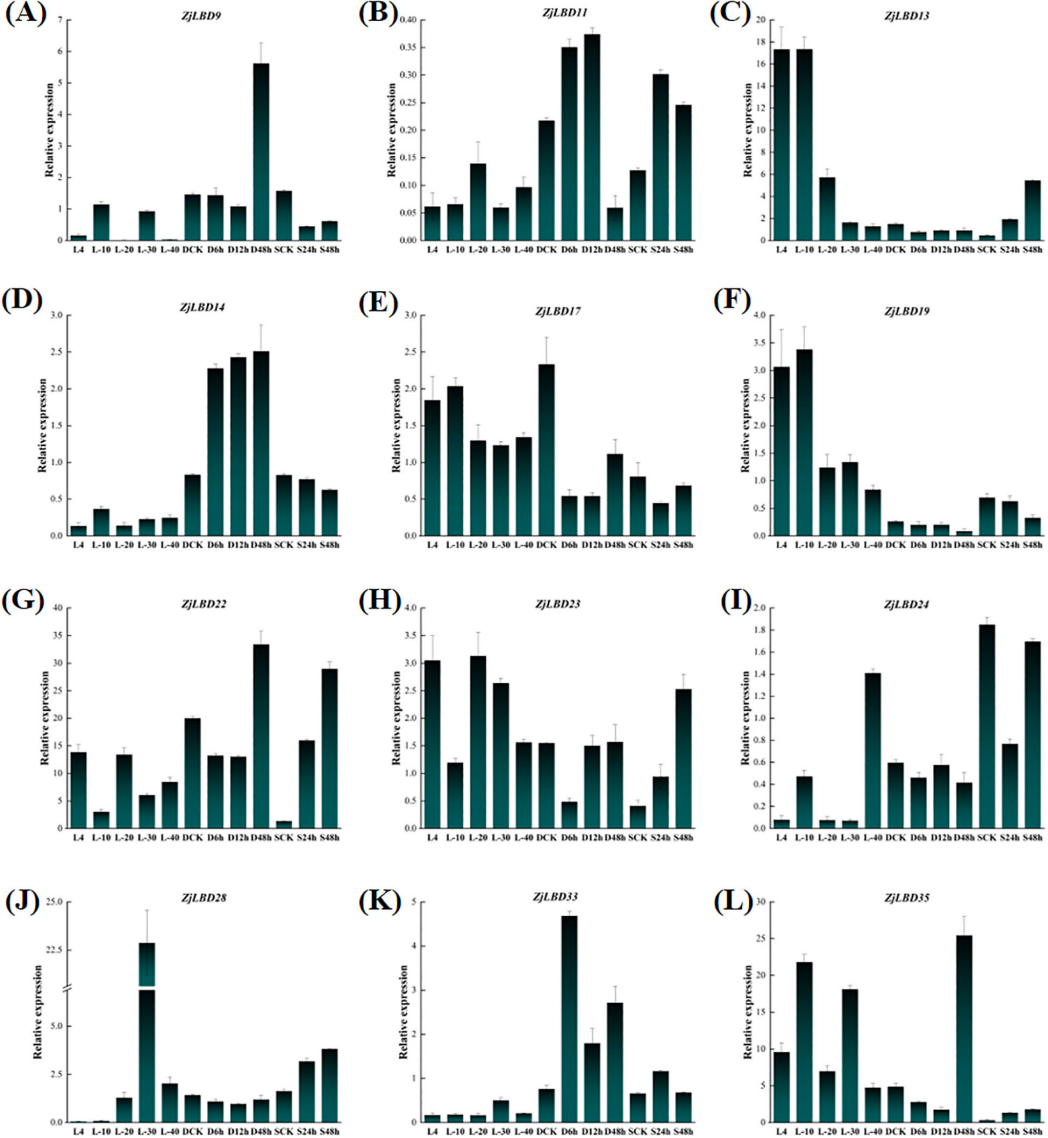
Expression pattern of *ZjLBD9* **(A)**, *ZjLBD11* **(B)**, *ZjLBD13* **(C)**, *ZjLBD14* **(D)**, *ZjLBD17* **(E)**, *ZjLBD19* **(F)**, *ZjLBD22* **(G)**, *ZjLBD23* **(H)**, *ZjLBD24* **(I)**, *ZjLBD28* **(J)**, *ZjLBD33* **(K)** and *ZjLBD35* **(L)** under low temperature, drought, and salt stress in sour jujube. L4, L-10, L-20, L-30, and L-40 represent sour jujube branches treated at 4 °C, -10 °C, -20 °C, -30 °C, and -40 °C. D6h, D12h and D48h represent the sour jujube leaves after drought treatment for 6 h, 12 h and 48 h. S24h, S48h represent the sour jujube leaves after 24 h and 48 h of NaCl treatment.

Based on the analysis of promoter and upstream transcription factor functions, 12 *ZjLBD* genes—*ZjLBD9*, *ZjLBD11*, *ZjLBD13*, *ZjLBD14*, *ZjLBD17*, *ZjLBD19*, *ZjLBD22*, *ZjLBD23*, *ZjLBD24*, *ZjLBD28*, *ZjLBD33* and *ZjLBD35*—from six subgroups were selected for analysis of their expression levels under three independent stress treatments: low temperature, drought, and salt conditions (**Figures 8A–L**). The results indicated that, except for *ZjLBD14*, the other *ZjLBD* genes exhibited distinct expression patterns under different low-temperature environments. Specifically, *ZjLBD22* was down-regulated at -10°C, with a 3.5-fold decrease compared to its expression at 4°C, while *ZjLBD9* showed a 6-fold increase at -10°C relative to at 4°C. At -30°C, *ZjLBD28* exhibited an approximately 383-fold increase in expression compared to 4°C, whereas *ZjLBD13* showed a 9-fold decrease. Furthermore, *ZjLBD19* expression was downregulated at -20°C and remained low even at -40°C. Under drought stress, the expression level of *ZjLBD14* remains significantly higher for 48 h of drought treatment than that under normal conditions. The expression level of *ZjLBD23* significantly decreased by 2-fold within 6 h of drought stress but returned to normal levels thereafter. Meanwhile, the expression of *ZjLBD33* was upregulated by 5-fold after 6 h of drought treatment. Additionally, after 48 h of drought treatment, two genes—*ZjLBD9* and *ZjLBD35*—showed significant upregulation compared to the control group, with increases of 3-fold and 4-fold, respectively. Meanwhile, *ZjLBD11* exhibited a significant reduction. In response to salt stress, the expression levels of *ZjLBD13*, *ZjLBD22*, *ZjLBD23*, *ZjLBD24*, *ZjLBD28*, and *ZjLBD35* gradually increased with the duration of salt stress. Notably, after 48 h of salt stress, the expression level of *ZjLBD22* was 21-fold that of the control group. Specifically, the expression of *ZjLBD9* was approximately halved after 24 h of salt stress compared to the untreated control group. The expression patterns of these genes reveal their regulatory function under different stress conditions, providing important clues for understanding gene functions.

## Discussion

The LBD family is integral to the regulation of tissue development as well as responses to environmental stimuli in plants. In sour jujube, we identified 37 *ZjLBD*s, fewer than the 43 in *Arabidopsis thaliana*, 58 in apple, and 57 in poplar. This suggests that the *ZjLBD* family has not experienced significant gene expansion events. The classification of LBD proteins into two major groups and six subgroups, as observed in sour jujube, aligned with patterns seen in *Arabidopsis* and poplar, indicating a conserved evolutionary origin of *LBD* gene family across different plant species. Based on the analysis of protein structural domains, the GAS block domain in ZjLBDs contained conserved proline and glycine residues. Lee et al. (2013) demonstrated that the conserved proline residue in the LOB domain of AtLBD18 is critical for DNA binding and biological function and substituting this proline residue with a leucine residue inhibited lateral root growth in *Arabidopsis* overexpressing *AtLBD16* and *AtLBD18*. These findings suggest the important role of the conserved proline residue in sour jujube. However, the function of the glycine residues remains unclear. The glycine residue, while conserved, may play a structural role in maintaining the stability of the GAS block domain. Motifs usually refer to short sequences involved in important biological processes. The presence of motifs 5–10 only in Class I; and motifs 3 and 4 only in Class II suggests they may have unique biological functions that remain to be determined. Additionally, we identified 15 segmental duplication events and 11 pairs of *ZjLBD* genes under purifying selection. These findings suggest that these *ZjLBD* genes may play important roles during evolution. Future studies should investigate the specific roles of these genes in environmental adaptation and their potential applications in breeding programs.

The structure of gene promoters and their regulatory pathways are closely related to many plant traits. The promoters of *ZjLBD* genes contain a variety of cis-regulatory elements with core physiological functions, suggesting that *ZjLBD* genes may respond to multiple internal and external environmental signals. Previous studies have shown that the expression of *ZjLBD* genes in plants is regulated by drought and ABA. For example, the overexpression of *PheLBD29* can enhance the drought tolerance of bamboo (Wu et al., 2023); AtLBD14 is involved in ABA-mediated lateral root formation and control in *Arabidopsis* (Jeon et al., 2017), and *ZmLBD5* negatively regulates maize drought tolerance by impairing ABA synthesis (Feng et al., 2022). Drought-inducible elements and ABRE elements coexist in the promoters of 14 *ZjLBD* genes, which may be involved in ABA-mediated drought stress responses. Furthermore, 11 *ZjLBD* genes contain both auxin-responsive elements and development-related elements, implying their roles in auxin signal transduction and tissue development. A similar result was found in *Medicago truncatula*, where MtARF2 binds to the auxin-responsive element in the *MtLBD17/29a* promoter to control nodulation and root architecture (Kirolinko et al., 2024). Additionally, the gene response to the environment may be closely related to the abundance of functional cis-acting elements. The promoters of *ZjLBD* genes have the highest number of TATA-box elements, indicating their key role in initiation of transcription (Jores et al., 2021). A study found that *SlLBD40*, which participates in jasmonic acid signal transduction during drought resistance in tomato, is a negative regulator of drought tolerance (Liu et al., 2020a). The promoter of *ZjLBD11* is rich in jasmonic acid response elements, and its expression was significantly downregulated after 48 h of drought stress, indicating that *ZjLBD11* may also act as a negative regulator of drought resistance in sour jujube.

A genome-wide study of LBD TFs in sour jujube provides valuable insights into the functions of these genes in specific pathways. Transcriptome data reveal that 11 *ZjLBD* genes are involved in flower development, while 7 *ZjLBD* genes are associated with root development. As shown in transgenic *Arabidopsis*, overexpression of *CsLBD37* leads to shorter plants, earlier flowering, and reduced seed production (Teng et al., 2022). *LBD13*, which is expressed in the meristems of lateral roots and elongated lateral roots, regulates lateral root formation (Cho et al., 2019). These findings suggest that *LBDs* in the same subgroup (Ib and IIa) in sour jujube may play crucial roles in regulating flowering time and lateral root development. The high expression of *ZjLBD23* from subgroup IIa in flowers indicates its potential functional roles during flower development. *ZjLBD11*, *ZjLBD13*, *ZjLBD22* and *ZjLBD28* are highly expressed in roots, indicating their potential involvement in the lateral root formation. Among these, *ZjLBD13* is gradually upregulated upon salt stress treatment, indicating that it may play a role in responding to salt stress through lateral root development (Zhang et al., 2024). Eight *ZjLBD* genes exhibit elevated expression levels during the white mature stage of fruit development. Specifically, the high expression fold of *ZjLBD23* at this stage compared to the semi-red stage suggests its role during the development of white mature fruits (Chen et al., 2017).

The potential upstream transcription factors of *ZjLBD*s were significantly enriched in response to stimuli, including responses to hormones, osmotic stress, and salt stress, suggesting that *ZjLBD*s may play an important role in environmental stress response. Notably, the functions of 9 *AtLBD* genes in this subgroup under low-temperature conditions have not been previously reported, and the potential upstream transcription factors of *ZjLBD*s may also be involved in xylem development, suggesting their potential unique roles in adapting to low-temperature stress in woody plants. *ZjLBD19*, which was differentially expressed at -20°C, -30°C and -40°C, contains a low-temperature responsive element, indicating its potential involvement in cold tolerance. The expression patterns of *ZjLBD13* and *ZjLBD28* suggest their responses to extreme low temperatures at -30°C, highlighting their significant functions in the cold tolerance of sour jujube. Under drought stress, *ZjLBD33*, which contains 3 drought-responsive elements, was differentially expressed. Furthermore, *ZjLBD14* and *ZjLBD35*, containing 3 and 6 ABRE elements, respectively, also showed differential expression after 12 h and 48 h of drought stress, indicating their roles in drought resistance. Under salt stress, both *ZjLBD9* and *ZjLBD22*, which contain ABRE elements, exhibited significant differential expression. Notably, *ZjLBD9* also includes 2 stress-responsive elements. These findings suggest that *ZjLBD9* and *ZjLBD22* may play crucial roles in the response to salt stress. Overall, the differential expression of these *ZjLBD* genes under various abiotic stress conditions highlights their vital contributions to stress resistance of sour jujube, providing valuable insights into the molecular mechanisms for plant adaptation to adverse environments.

## Conclusion

In this study, we identified 37 ZjLBD TFs in sour jujube and classified them into two groups and six subgroups based on their evolutionary relationships. These *ZjLBD* genes have undergone 15 segmental duplication events, and 13 genes show evidence of purifying selection. All identified proteins contain the conserved CX2CX6CX3C motif, which is essential for DNA binding. Class I proteins feature the LX6LX3LX6L motif, associated with protein dimerization, and subgroups Ia, Ib, and Ic contain the GAS domain, which is crucial for biological functions. Gene structure analysis revealed that *ZjLBD* genes from different groups have distinct intron lengths and motif sequence types. Promoter and upstream TF prediction analysis suggested that *ZjLBD* genes promoters may be involved in pathways related to growth and development, stress responses, and hormone signaling. Expression analysis indicated that several *ZjLBD* genes play roles in flower, root and fruit development. For example, *ZjLBD23* may be involved in flower and fruit development during the white mature stage, while *ZjLBD11*, *ZjLBD13*, *ZjLBD22* and *ZjLBD28* may associated with the lateral root formation. These genes also exhibit potential functions under stress conditions. Specifically, *ZjLBD13*, *ZjLBD19* and *ZjLBD28* showed differential expression in response to extreme low-temperature conditions, *ZjLBD11*, *ZjLBD14*, *ZjLBD33* and *ZjLBD35* may respond to 48 h of drought stress, and *ZjLBD9*, *ZjLBD13* and *ZjLBD22* exhibited differential expression after 48 h of salt stress. However, the regulatory roles of these ZjLBD TFs in sour jujube development and stress response remain to be confirmed in future studies.

## Data Availability

The original contributions presented in the study are included in the article/**Supplementary Material**. Further inquiries can be directed to the corresponding authors.
