# Supplementary material for: Genome-wide characterization and expression analysis of LBD transcription factors in Ziziphus jujuba var. spinosa: putative roles in tissue development and abiotic stress adaptation
Source: Front Plant Sci. 2025 May 21;16:1602440. doi: 10.3389/fpls.2025.1602440 (PMC12133987; doi:10.3389/fpls.2025.1602440)
Supplement: Supplementary file 1 [file Table1.docx]

| **Gene name** | **Forward** | **Reverse** |
| --- | --- | --- |
| *ZjActin* | AGCCTTCCTGCCAACGAGT | TTGCTTCTCACCCTTGATGC |
| *ZjLBD7* | GGAGATGCGCCGATAAATGC | CGTACACCATGCTGGTCACT |
| *ZjLBD9* | GTACGGTTGCATGGGGGTAA | AGCAATGGAGACCACACCAG |
| *ZjLBD11* | GAGTCTCAGAGAGCGGATGC | TGCTGGCATTGCATGTTCAC |
| *ZjLBD13* | CGCTTCATGCAAGTTGCTCC | TGAACCGGAAGCTCCTGAAG |
| *ZjLBD14* | CCCCAGGAACCCTAAACACC | TTTGACTCTCCGGCACTTCC |
| *ZjLBD17* | CAACGCCACTGTTTTCCTCG | CCAACTACCGGACCACAACA |
| *ZjLBD19* | CACTTGCACAAGCTGAGGTG | TGACCACAAGGACTCTCCCA |
| *ZjLBD22* | CTTTACGATGCTTGCGGTCG | GGCGAATATCGCAGGCTTTG |
| *ZjLBD23* | TTCTGAGGTCGTCACTGCAC | CCTACAGCTCCGTTCACTGG |
| *ZjLBD24* | GTTGGTGGCTGTCACCGTAT | ATGATGAGTCCCAACAGGCG |
| *ZjLBD28* | TCCGCTCACAATAGCAGACC | CCTGATGATGAGCCTGTGCT |
| *ZjLBD33* | GCCCTGACAATGCGAATCAG | TCACAACTCCCAACAAGGTT |
| *ZjLBD35* | CGCAGCCTTTCTCTCAATGC | CCCATCCGAATCCACCGAAT |

Supplementary Table 1 Primers used in the study
