# Supplementary material for: Genome-wide characterization and expression analysis of LBD transcription factors in Ziziphus jujuba var. spinosa: putative roles in tissue development and abiotic stress adaptation
Source: Front Plant Sci. 2025 May 21;16:1602440. doi: 10.3389/fpls.2025.1602440 (PMC12133987; doi:10.3389/fpls.2025.1602440)
Supplement: Supplementary file 2 [file Table2.docx]

Supplementary Table 2 Selective pressure analysis of *ZjLBD* genes

| Sequence 1 | Ka | Ks | Ka/Ks | Selection pressure |
| --- | --- | --- | --- | --- |
| ZjLBD1/ ZjLBD18 | 0.679789 | NaN | NaN | uncertain |
| ZjLBD5/ ZjLBD24 | 0.455946 | 1.319092 | 0.345651 | purifying selection |
| ZjLBD5/ ZjLBD26 | 0.77369 | NaN | NaN | uncertain |
| ZjLBD7/ ZjLBD11 | 0.190765 | 1.330715 | 0.143355 | purifying selection |
| ZjLBD8/ ZjLBD13 | 0.136645 | 0.876551 | 0.15589 | purifying selection |
| ZjLBD9/ ZjLBD14 | 0.139508 | 1.755481 | 0.07947 | purifying selection |
| ZjLBD15/ ZjLBD20 | 0.238131 | 1.442006 | 0.165138 | purifying selection |
| ZjLBD17/ ZjLBD35 | 0.330091 | 1.838092 | 0.179583 | purifying selection |
| ZjLBD22/ ZjLBD35 | 0.358324 | 1.26299 | 0.283711 | purifying selection |
| ZjLBD23/ ZjLBD34 | 0.362352 | 1.994004 | 0.181721 | purifying selection |
| ZjLBD23/ ZjLBD37 | 0.325169 | 1.647918 | 0.197321 | purifying selection |
| ZjLBD24/ ZjLBD26 | 0.856741 | 4.352495 | 0.196839 | purifying selection |
| ZjLBD34/ ZjLBD37 | 0.360299 | 1.55101 | 0.2323 | purifying selection |

NaN represent High Sequence Divergence Value (pS>=0.75).
